# Supplementary material for: Hot spot prediction in protein-protein interactions by an ensemble system
Source: BMC Syst Biol. 2018 Dec 31;12(Suppl 9):132. doi: 10.1186/s12918-018-0665-8 (PMC6311905; doi:10.1186/s12918-018-0665-8)
Supplement: Supplementary file 2 — All relevant MATLAB code. A simple Matlab implement of our predictor. (ZIP 19,742 kb) [file 12918_2018_665_MOESM2_ESM.zip › Additional file 2/Read ASA/ASA Property/SKEMPI/2J12A.txt]

 NetSurfP - Protein Surface Accessibility and Secondary Structure Predictions

  

|  |  |  |
| --- | --- | --- |
|  |  | NetSurfP - Protein Surface Accessibility and Secondary Structure Predictions  Technical University of Denmark |

  


---

```
# For publication of results, please cite:
# A generic method for assignment of reliability scores applied to solvent accessibility predictions.
# Bent Petersen, Thomas Nordahl Petersen, Pernille Andersen, Morten Nielsen and Claus Lundegaard
# BMC Structural Biology 2009, 9:51 doi:10.1186/1472-6807-9-51
#
# Column 1: Class assignment - B for buried or E for Exposed - Threshold: 25% exposure, but not based on RSA
# Column 2: Amino acid
# Column 3: Sequence name
# Column 4: Amino acid number
# Column 5: Relative Surface Accessibility - RSA
# Column 6: Absolute Surface Accessibility
# Column 7: Z-fit score for RSA prediction
# Column 8: Probability for Alpha-Helix
# Column 9: Probability for Beta-strand
# Column 10: Probability for Coil
E R  2J12A                  1    0.649 148.644   0.750   0.003   0.003   0.994
E T  2J12A                  2    0.282  39.086  -0.429   0.020   0.205   0.775
B L  2J12A                  3    0.177  32.354  -0.619   0.022   0.359   0.619
B W  2J12A                  4    0.145  34.921  -0.668   0.021   0.451   0.528
B T  2J12A                  5    0.261  36.228  -1.096   0.022   0.359   0.619
E T  2J12A                  6    0.348  48.323  -0.815   0.004   0.138   0.858
E P  2J12A                  7    0.468  66.466  -1.140   0.005   0.045   0.951
E D  2J12A                  8    0.574  82.699  -1.111   0.005   0.015   0.979
B T  2J12A                  9    0.296  41.125  -0.418   0.005   0.015   0.979
E S  2J12A                 10    0.467  54.744  -1.019   0.018   0.019   0.964
B P  2J12A                 11    0.271  38.441  -0.822   0.018   0.047   0.935
E N  2J12A                 12    0.380  55.617  -0.699   0.004   0.138   0.858
B C  2J12A                 13    0.048   6.767   0.135   0.004   0.420   0.576
E T  2J12A                 14    0.434  60.168   1.680   0.002   0.816   0.182
B I  2J12A                 15    0.118  21.830  -0.111   0.001   0.900   0.099
E A  2J12A                 16    0.475  52.323  -0.124   0.001   0.900   0.099
E Q  2J12A                 17    0.404  72.226  -0.516   0.003   0.718   0.279
E D  2J12A                 18    0.458  65.955  -0.308   0.004   0.197   0.799
E K  2J12A                 19    0.532 109.515  -0.443   0.005   0.045   0.951
E D  2J12A                 20    0.482  69.427  -0.384   0.005   0.045   0.951
B S  2J12A                 21    0.044   5.180   0.006   0.005   0.262   0.733
E K  2J12A                 22    0.254  52.186   1.387   0.003   0.718   0.279
B L  2J12A                 23    0.032   5.859   0.455   0.001   0.959   0.040
B T  2J12A                 24    0.056   7.739   0.452   0.001   0.959   0.040
B L  2J12A                 25    0.018   3.332   1.345   0.001   0.959   0.040
B V  2J12A                 26    0.030   4.626   0.191   0.001   0.959   0.040
B L  2J12A                 27    0.023   4.156   0.949   0.001   0.959   0.040
B T  2J12A                 28    0.049   6.782   0.178   0.001   0.900   0.099
B K  2J12A                 29    0.253  52.063   0.130   0.004   0.616   0.381
B C  2J12A                 30    0.197  27.715  -1.002   0.004   0.138   0.858
E G  2J12A                 31    0.415  32.629  -1.245   0.005   0.015   0.979
B S  2J12A                 32    0.207  24.272  -2.248   0.004   0.197   0.799
B Q  2J12A                 33    0.211  37.667   0.787   0.001   0.900   0.099
B I  2J12A                 34    0.052   9.528   0.044   0.001   0.959   0.040
B L  2J12A                 35    0.141  25.780   0.601   0.001   0.959   0.040
B A  2J12A                 36    0.048   5.268  -0.604   0.001   0.959   0.040
B N  2J12A                 37    0.138  20.159   0.235   0.001   0.959   0.040
B V  2J12A                 38    0.045   6.932   0.452   0.001   0.959   0.040
B S  2J12A                 39    0.068   7.923  -0.191   0.000   0.983   0.017
B L  2J12A                 40    0.054   9.942   0.334   0.000   0.983   0.017
B I  2J12A                 41    0.184  34.003   1.125   0.001   0.959   0.040
B V  2J12A                 42    0.062   9.576  -1.388   0.001   0.900   0.099
B V  2J12A                 43    0.162  24.976  -0.407   0.002   0.816   0.182
E A  2J12A                 44    0.413  45.568  -0.188   0.004   0.616   0.381
B G  2J12A                 45    0.282  22.178  -1.495   0.022   0.359   0.619
E K  2J12A                 46    0.489 100.670  -0.642   0.021   0.279   0.699
B Y  2J12A                 47    0.259  55.412  -1.233   0.021   0.279   0.699
E H  2J12A                 48    0.371  67.503  -0.393   0.074   0.484   0.442
E I  2J12A                 49    0.396  73.297   0.103   0.074   0.484   0.442
B I  2J12A                 50    0.096  17.741  -0.479   0.135   0.317   0.548
E N  2J12A                 51    0.377  55.120  -0.873   0.118   0.150   0.732
E N  2J12A                 52    0.602  88.089  -1.213   0.113   0.087   0.800
E K  2J12A                 53    0.481  99.045  -1.156   0.113   0.043   0.844
E T  2J12A                 54    0.420  58.212  -1.180   0.052   0.084   0.864
E N  2J12A                 55    0.548  80.271  -0.493   0.018   0.047   0.935
E P  2J12A                 56    0.430  61.088  -0.517   0.018   0.088   0.893
E K  2J12A                 57    0.552 113.608  -0.399   0.004   0.197   0.799
E I  2J12A                 58    0.452  83.601  -0.473   0.004   0.616   0.381
E K  2J12A                 59    0.325  66.811   0.324   0.003   0.718   0.279
E S  2J12A                 60    0.321  37.668   0.484   0.001   0.900   0.099
B F  2J12A                 61    0.053  10.717   0.719   0.001   0.959   0.040
B T  2J12A                 62    0.235  32.664   1.172   0.001   0.959   0.040
B I  2J12A                 63    0.024   4.403   1.090   0.000   0.983   0.017
B K  2J12A                 64    0.214  43.979   1.349   0.000   0.983   0.017
B L  2J12A                 65    0.034   6.262   0.595   0.001   0.959   0.040
B L  2J12A                 66    0.175  32.116   1.058   0.001   0.959   0.040
B F  2J12A                 67    0.040   8.008  -0.076   0.002   0.816   0.182
B N  2J12A                 68    0.235  34.360   0.564   0.019   0.141   0.840
E K  2J12A                 69    0.612 125.806  -1.149   0.018   0.047   0.935
E N  2J12A                 70    0.548  80.271   0.277   0.018   0.047   0.935
B G  2J12A                 71    0.137  10.790  -0.975   0.018   0.088   0.893
E V  2J12A                 72    0.325  49.968   0.557   0.021   0.451   0.528
B L  2J12A                 73    0.104  18.987   0.024   0.023   0.655   0.322
B L  2J12A                 74    0.204  37.426   0.295   0.022   0.552   0.426
E D  2J12A                 75    0.513  73.952  -0.938   0.021   0.279   0.699
E N  2J12A                 76    0.550  80.447  -1.592   0.018   0.088   0.893
B S  2J12A                 77    0.325  38.078  -1.685   0.018   0.047   0.935
E N  2J12A                 78    0.489  71.546  -1.755   0.018   0.088   0.893
B L  2J12A                 79    0.207  37.828  -0.974   0.018   0.088   0.893
E G  2J12A                 80    0.514  40.428  -0.933   0.052   0.084   0.864
E K  2J12A                 81    0.569 117.002  -0.461   0.052   0.084   0.864
E A  2J12A                 82    0.602  66.340  -1.830   0.052   0.084   0.864
E Y  2J12A                 83    0.340  72.594  -0.481   0.064   0.216   0.721
B W  2J12A                 84    0.160  38.432  -0.061   0.066   0.296   0.638
E N  2J12A                 85    0.345  50.449  -1.071   0.069   0.386   0.545
B F  2J12A                 86    0.234  46.904  -0.419   0.069   0.386   0.545
E R  2J12A                 87    0.338  77.471   0.189   0.069   0.386   0.545
E S  2J12A                 88    0.442  51.849  -1.263   0.064   0.216   0.721
E G  2J12A                 89    0.580  45.622  -1.566   0.018   0.088   0.893
E N  2J12A                 90    0.406  59.409  -1.583   0.018   0.088   0.893
E S  2J12A                 91    0.328  38.395  -1.839   0.020   0.205   0.775
E N  2J12A                 92    0.398  58.267  -1.457   0.021   0.279   0.699
E V  2J12A                 93    0.352  54.133  -0.663   0.064   0.216   0.721
E S  2J12A                 94    0.460  53.935  -1.822   0.056   0.142   0.802
E T  2J12A                 95    0.395  54.787  -1.146   0.113   0.087   0.800
E A  2J12A                 96    0.380  41.854  -0.889   0.113   0.087   0.800
B Y  2J12A                 97    0.263  56.246  -0.997   0.118   0.150   0.732
E E  2J12A                 98    0.356  62.158  -0.923   0.118   0.150   0.732
E K  2J12A                 99    0.350  71.913  -0.551   0.118   0.150   0.732
B A  2J12A                100    0.159  17.511  -0.345   0.135   0.317   0.548
B I  2J12A                101    0.180  33.318  -0.470   0.069   0.386   0.545
B G  2J12A                102    0.237  18.660  -0.871   0.074   0.484   0.442
B F  2J12A                103    0.066  13.266  -1.302   0.066   0.296   0.638
B M  2J12A                104    0.091  18.229   0.519   0.019   0.141   0.840
B P  2J12A                105    0.129  18.291  -0.732   0.018   0.088   0.893
E N  2J12A                106    0.301  44.037  -0.957   0.018   0.088   0.893
B L  2J12A                107    0.244  44.658  -0.705   0.020   0.205   0.775
E V  2J12A                108    0.345  53.057  -0.873   0.022   0.359   0.619
B A  2J12A                109    0.094  10.403  -1.119   0.021   0.451   0.528
B Y  2J12A                110    0.151  32.226   0.449   0.022   0.359   0.619
B P  2J12A                111    0.143  20.334  -1.244   0.020   0.205   0.775
E K  2J12A                112    0.385  79.215   0.569   0.018   0.088   0.893
E P  2J12A                113    0.349  49.566  -0.811   0.018   0.088   0.893
E S  2J12A                114    0.581  68.046  -0.657   0.018   0.047   0.935
E N  2J12A                115    0.614  89.963  -1.362   0.018   0.047   0.935
E S  2J12A                116    0.566  66.300  -1.332   0.018   0.088   0.893
E K  2J12A                117    0.546 112.230  -0.069   0.022   0.359   0.619
E K  2J12A                118    0.546 112.312  -0.227   0.021   0.451   0.528
E Y  2J12A                119    0.378  80.885   0.253   0.022   0.359   0.619
B A  2J12A                120    0.261  28.795  -1.432   0.021   0.279   0.699
E R  2J12A                121    0.516 118.187   0.203   0.005   0.262   0.733
E D  2J12A                122    0.266  38.403  -1.008   0.004   0.616   0.381
B I  2J12A                123    0.167  30.950   0.796   0.001   0.900   0.099
B V  2J12A                124    0.058   8.868  -0.095   0.001   0.959   0.040
B Y  2J12A                125    0.077  16.540   0.061   0.001   0.959   0.040
B G  2J12A                126    0.040   3.156  -1.540   0.001   0.959   0.040
B T  2J12A                127    0.070   9.751   0.374   0.001   0.959   0.040
B I  2J12A                128    0.030   5.457   0.497   0.001   0.959   0.040
B Y  2J12A                129    0.087  18.549   0.535   0.001   0.959   0.040
B L  2J12A                130    0.114  20.818  -0.089   0.002   0.816   0.182
E G  2J12A                131    0.367  28.891   0.248   0.005   0.336   0.660
E G  2J12A                132    0.372  29.237  -1.415   0.004   0.085   0.910
E K  2J12A                133    0.517 106.409   0.016   0.004   0.138   0.858
E P  2J12A                134    0.517  73.306  -0.873   0.005   0.045   0.951
E D  2J12A                135    0.608  87.613  -1.384   0.005   0.015   0.979
E Q  2J12A                136    0.329  58.688   0.647   0.004   0.085   0.910
B P  2J12A                137    0.193  27.401  -0.358   0.005   0.336   0.660
B A  2J12A                138    0.058   6.392   0.584   0.001   0.900   0.099
E V  2J12A                139    0.245  37.626   0.944   0.001   0.959   0.040
B I  2J12A                140    0.034   6.253   0.250   0.001   0.959   0.040
B K  2J12A                141    0.180  37.108   0.975   0.001   0.959   0.040
B T  2J12A                142    0.028   3.828   0.387   0.001   0.959   0.040
B T  2J12A                143    0.125  17.268  -0.050   0.001   0.959   0.040
B F  2J12A                144    0.077  15.534   0.153   0.001   0.900   0.099
B N  2J12A                145    0.199  29.148  -0.846   0.004   0.514   0.481
E Q  2J12A                146    0.474  84.639  -0.691   0.004   0.138   0.858
E E  2J12A                147    0.510  89.097  -1.081   0.004   0.138   0.858
E T  2J12A                148    0.348  48.226  -0.710   0.004   0.085   0.910
E G  2J12A                149    0.641  50.455  -0.681   0.005   0.045   0.951
B C  2J12A                150    0.184  25.806  -0.564   0.005   0.262   0.733
B E  2J12A                151    0.155  26.991  -1.166   0.004   0.616   0.381
B Y  2J12A                152    0.132  28.208   0.255   0.002   0.816   0.182
B S  2J12A                153    0.087  10.196  -1.153   0.001   0.959   0.040
B I  2J12A                154    0.057  10.600   0.592   0.001   0.959   0.040
B T  2J12A                155    0.200  27.671   0.547   0.001   0.959   0.040
B F  2J12A                156    0.064  12.805   0.416   0.001   0.959   0.040
E N  2J12A                157    0.320  46.819   0.957   0.001   0.959   0.040
B F  2J12A                158    0.099  19.950   0.489   0.001   0.959   0.040
E S  2J12A                159    0.344  40.340   0.449   0.001   0.900   0.099
B W  2J12A                160    0.267  64.238  -1.011   0.022   0.552   0.426
E S  2J12A                161    0.374  43.891  -1.159   0.019   0.141   0.840
E K  2J12A                162    0.548 112.621  -1.185   0.018   0.088   0.893
E T  2J12A                163    0.596  82.679  -0.243   0.021   0.279   0.699
E Y  2J12A                164    0.278  59.344   0.280   0.021   0.279   0.699
E E  2J12A                165    0.572  99.876   0.088   0.004   0.138   0.858
E N  2J12A                166    0.668  97.795  -0.216   0.004   0.085   0.910
E V  2J12A                167    0.346  53.196   0.663   0.004   0.197   0.799
E E  2J12A                168    0.551  96.347   0.838   0.021   0.451   0.528
B F  2J12A                169    0.102  20.512  -0.079   0.021   0.451   0.528
E E  2J12A                170    0.585 102.252  -0.669   0.021   0.279   0.699
B T  2J12A                171    0.181  25.091  -0.211   0.004   0.085   0.910
E T  2J12A                172    0.371  51.430  -0.759   0.004   0.085   0.910
B S  2J12A                173    0.264  30.917  -1.149   0.004   0.197   0.799
B F  2J12A                174    0.073  14.671  -0.195   0.004   0.616   0.381
B T  2J12A                175    0.142  19.626  -0.719   0.002   0.816   0.182
B F  2J12A                176    0.053  10.657   0.394   0.001   0.900   0.099
B S  2J12A                177    0.108  12.693  -0.433   0.001   0.900   0.099
B Y  2J12A                178    0.098  20.857   0.219   0.001   0.900   0.099
B I  2J12A                179    0.110  20.387  -0.672   0.002   0.816   0.182
B A  2J12A                180    0.147  16.232  -0.840   0.003   0.718   0.279
E Q  2J12A                181    0.363  64.814  -0.202   0.004   0.514   0.481
E E  2J12A                182    0.818 142.835   0.482   0.016   0.005   0.979

Explain the output.  Go back.
```

---
